# Mucosal Progranulin expression is induced by *H. pylori*, but independent of Secretory Leukocyte Protease Inhibitor (SLPI) expression

**DOI:** 10.1186/1471-230X-11-63

**Published:** 2011-05-26

**Authors:** Thomas Wex, Doerthe Kuester, Cornelius Schönberg, Daniel Schindele, Gerhard Treiber, Peter Malfertheiner

**Affiliations:** 1 Department of Gastroenterology, Hepatology and Infectious Diseases, Otto-von-Guericke University, Leipziger Str. 44, Magdeburg, D-39120, Germany; 2 Institute of Pathology, Otto-von-Guericke University, Leipziger Str. 44, Magdeburg, D-39120, Germany; 3 Department of Gastroenterology, Oncology and General Internal Medicine, Zollernalb Clinic, 72336 Balingen, Germany

## Abstract

**Background:**

Mucosal levels of Secretory Leukocyte Protease Inhibitor (SLPI) are specifically reduced in relation to *H. pylori*-induced gastritis. Progranulin is an epithelial growth factor that is proteolytically degraded into fragments by elastase (the main target of SLPI). Considering the role of SLPI for regulating the activity of elastase, we studied whether the *H. pylori*-induced reduction of SLPI and the resulting increase of elastase-derived activity would reduce the Progranulin protein levels both *ex vivo *and *in vitro*.

**Methods:**

The expression of Progranulin was studied in biopsies of *H. pylori*-positive, -negative and -eradicated subjects as well as in the gastric tumor cell line AGS by ELISA, immunohistochemistry and real-time RT-PCR.

**Results:**

*H. pylori*-infected subjects had about 2-fold increased antral Progranulin expression compared to *H. pylori*-negative and -eradicated subjects (P < 0.05). Overall, no correlations between mucosal Progranulin and SLPI levels were identified. Immunohistochemical analysis confirmed the upregulation of Progranulin in relation to *H. pylori *infection; both epithelial and infiltrating immune cells contributed to the higher Progranulin expression levels. The *H. pylori*-induced upregulation of Progranulin was verified in AGS cells infected by *H. pylori*. The down-regulation of endogenous SLPI expression in AGS cells by siRNA methodology did not affect the Progranulin expression independent of the infection by *H. pylori*.

**Conclusions:**

Taken together, Progranulin was identified as novel molecule that is upregulated in context to *H. pylori *infection. In contrast to other diseases, SLPI seems not to have a regulatory role for Progranulin in *H. pylori*-mediated gastritis.

## Background

Secretory leukocyte protease inhibitor (SLPI) is a member of the chelonianin class of serine protease inhibitors, and is predominantly expressed in secretory epithelial cells of mucosal surfaces, immune cells and has been identified in various tissues [1-3]. Among serine proteinase inhibitors, SLPI is considered as "alarm proteinase inhibitor" that is upregulated during infection or inflammation to compensate for high human neutrophil elastase [1,4]. The C-terminus of SLPI primarily inhibits human elastase, but is capable of inhibiting other serine proteinases such as tryptase and cathepsin G [5]. In addition to its function as an antiprotease, SLPI possesses antimicrobial activity against several bacteria and fungi [1,4,6]. Furthermore, it was shown that SLPI controls cell proliferation by regulation of growth-associated genes such as cyclin D and transforming growth factor (TGF)-β1 [7], modifies the activation of macrophages [4] and regulates the LPS-induced activation of the transcription factor "nuclear factor kappa B" (NF-κB) [8,9]. SLPI-deficient mice provided evidence for functional involvement of SLPI in wound healing [10] and lipopolysaccharide (LPS)-mediated inflammation [11]. In context to its role as "alarm proteinase inhibitor", SLPI was found to be differentially regulated in inflammatory diseases and cancer. Increased expression or elevated serum levels of SLPI were reported in human sepsis and experimental endotoxemia [12], febrile patients [13], Wegners's granulomatosis [14], gastric cancer [15] and pulmonary infection [16]. In contrast, other bacterial or viral infections in lung [17], stomach [18] and cervical epithelial cells [19] were found to be associated with decreased SLPI levels. The underlying mechanisms responsible for the different regulation of SLPI have not been identified, but most likely both microbial and host factors contribute to the up- or downregulation of SLPI in the various diseases. Notably, the reduction of SLPI levels correlated inversely with the severity of inflammation (infiltration of granulocytes) and neutrophil elastase activity in the gastric mucosa of *H. pylori-*infected individuals [20,21] and the bronchoalveolar lavage fluid (BALF) of *Pseudomonas*-infected subjects [17].

Progranulin, also known as acrogranin, proepithelin and PC cell derived-growth factor, is a 68 kDa glycoprotein secreted by many epithelial and immune cells [22]. The full-length protein is subsequently modified by limited proteolysis leading to the generation of 6-25 kDa fragments called granulins [23]. Pathophysiologically, Progranulin has drawn a lot of attention in the last years since it has been identified that mutations of the corresponding *granulin *gene are causally linked to the development of frontotemporal dementia [24]. Individuals with these mutations exhibit tau-negative, but ubiquitin-positive, inclusions in their brain that eventually cause frontotemporal dementia. Both the precursor (Progranulin) and the degraded forms (Granulins) mediate different cellular effects in a variety of pathophysiological conditions such as inflammation, proliferation, carcinogenesis and wound healing [25]. While Progranulin acts as growth factor for epithelial cells, fibroblasts and neurons and has anti-inflammatory properties [26,27], granulins drive inflammation leading to the infiltration of immune cells and induced cytokine expression [28,29]. The conversion of Progranulin to granulins, which is the critical step in the regulation of the balance between both molecular forms, is controlled by SLPI that binds Progranulin and prevents degradation by elastase [23]. The importance of this interaction for the wound healing was demonstrated at the SLPI-deficient mice [10]. The lack of SLPI resulted in higher serine protease-derived activities that were associated with impaired wound healing in these animals [10]. The delayed wound healing was normalized after the addition of Progranulin providing evidence for the importance of the interaction between Progranulin and SLPI.

We recently identified a marked down-regulation of mucosal SLPI levels in *H. pylori*-infected subjects [18]. The role of SLPI for the balance between Progranulin and granulins and the high prevalence of mucosal injuries (ulcer, erosions) in *H. pylori*-infected subjects, prompted us to study the expression levels of Progranulin in context to that of SLPI in relation to *H. pylori *status. Considering the role of SLPI for regulating the activity of elastase, we hypothesized that the *H. pylori*-induced reduction of SLPI would lead to a reduction of mucosal Progranulin levels, since the higher elastase activities in the mucosa of *H. pylori*-infected subjects would degrade the molecule into the granulin fragments. In addition, gastric epithelial cells (either infected with *H. pylori *± transfection of SLPI-siRNA) were used as *in vitro *model to prove the proposed hypothesis.

## Methods

### Study design and H. pylori status

The study protocol was conducted according to the declaration of Helsinki and approved by the ethics' committee of the Otto-von-Guericke University (No. of ethical vote: 143/99) as well as government authorities; all participants signed informed consent before entering the study. Details of the protocol (inclusion, exclusion criteria, and demographic data) were described previously [20]. The initial protocol was aimed at studying the interaction of *H. pylori *infection and low-dose aspirin. Briefly, human healthy volunteers were stratified according to the *H. pylori *status leading to the *H. pylori*-positive (*H. pylori*^*+*^, n = 10) and -negative (*H. pylori*^-^, n = 10) group. After successful eradication therapy, 9 out of 10 *H. pylori*-infected individuals agreed to participate in this study after 3 months again composing the *H. pylori*-eradicated (*H. pylori*^*erad*^) group. In order to investigate the potential interaction between Progranulin and SLPI, mucosal and serum levels as well as gene expression levels of Progranulin were analyzed retrospectively in existing samples and tissue specimens in relation to *H. pylori *status and SLPI expression levels published previously [20]. The analysis of Progranulin expression was performed in the "pre-treatment" samples, which correspond to day 0 [20] before "low-dose" aspirin was taken by the individuals.

The study includes a correlation analysis of mucosal Progranulin levels with those of SLPI studied in the same cohorts previously; details concerning the analysis of SLPI were reported recently [20].

### Determination of Progranulin expression quantitative RT-PCR and ELISA

Progranulin levels were quantified in the total protein extract from mucosal biopsies at sera stored at -80°C in previous study [22]. Progranulin levels were quantified using the Progranulin ELISA kit (Axxora GmbH, Lörrach, Germany; No: AG-45A-0018PP-KI01) as described by the manufacturer. Protein levels were normalized to ng/μg total protein content of extracted mucosal samples or ng/ml for sera.

Corresponding Progranulin m-RNA levels were determined by quantitative RT-PCR using existing cDNA samples stored at -80°C. Quantitative RT-PCR was performed using an iCycler (BioRad, Munich, Germany) and HotStarTaq Master Mix™ (Qiagen, Hilden, Germany) as described [23]. Initial activation of Taq-polymerase at 95°C for 15 min was followed by 40 cycles with denaturation at 94°C for 30 s, annealing at 60°C for 30 s and elongation at 72°C for 30 s. The fluorescence intensity of the double-strand specific SYBR-Green I, reflecting the amount of actually formed PCR-product, was read real-time at the end of each elongation step. Then specific initial template mRNA amounts were calculated by determining the time point at which the linear increase of sample PCR product started, relative to the corresponding points of a standard curve; these are given as arbitrary units (a.u.). Both PCR products were cloned into the pDIRECT™ (Qiagen, Hilden, Germany), and subsequent dilutions of the corresponding plasmid DNA were used to create a standard curve for the RT-PCR. The correlation coefficients of both Progranulin and β-actin standards were > 0.95. β-actin mRNA amounts were used to normalize the cDNA contents of the different samples. Final data reflect the ratio in a.u. between Progranulin transcript and β-actin transcript levels. The following primers were used for the RT-PCR analysis: ß-actin (fw:5'-GCC-ATC-CTG-CGT-CTG-GAC-C-3' rev: 5'-ACA-TGG-TGG-TGC-CGC-CAG-ACA-G-3'; 400 bp), and Progranulin (fw:5`-ATG-GCC-CAC-AAC-ACT-GAG-CAG-G-3`, rev: 5`-TCT-GGG-CAG-GGA-GCT-TCT-TTG-C-3`, 440 bp). Both cDNA fragments included intron-spanning regions resulting in the generation of a larger PCR product from genomic DNA or its exclusion. Therefore, all identified PCR products can exclusively be attributed to the mRNA pool of the sample.

### Immunohistochemical analysis of Progranulin expression in the gastric mucosa

To study the cellular origin of Progranulin expression in antral and corpus mucosa, tissue specimens from all 29 individuals were subjected to immunohistochemical analysis. The pathologist (D.K.) was blinded to the group assignment of samples. Paraffin-embedded biopsy specimens were cut into 3 μm thick sections, mounted on glass slides, and treated with Xylol and dehydrated by standard protocols. For antigen retrieval, specimens were boiled three times in 0.01 M sodium citrate puffer (pH 6.0) for 10 min in a microwave (600W). Incubation with primary polyclonal goat-derived anti-Progranulin antibody (BAF2420, dilution 1:1.000, R&D Systems, Minneapolis, MN, USA) was conducted at 37°C for 35 min and followed by PBS-washing. Positive immunohistochemical reactions were revealed using the iVIEWTM DAB Detection Kit (Ventana, Germany) as chromogen substrate. Finally, the samples were counter-stained with hematoxilin, dehydrated and mounted using DEPEX (Serva, Heidelberg, Germany). For positive control normal prostate tissue was used. For negative control corresponding stainings were performed using unrelated goat antiserum that did not lead to a specific staining (data not shown).

Expression of Progranulin was scored for the epithelium of the mucosal surface and gastric glands of the antrum and corpus in 3 representative high power fields (Zeiss Axioskop 50). Staining intensity (SI) and the percentage of positive cells (PP) were assessed using the following semiquantitative score: SI was classified in 0 (no staining), 1 (weak), 2 (moderate) and 3 (strong); PP: 0 (no positive cells), 1 (< 10%), 2 (10-50%), 3 (51 - 80%), 4 (> 80%). For each slide the immunoreactive score (IRS) was calculated as [SI x PP] with a possible maximum score of 12. Immunohistochemical expression of Progranulin was separately scored for surface epithelium and glands, and then these scores were summarized as "total score" that were statistically analyzed among the three groups. The maximum score for epithelial expression of Progranulin was "24". Since all type of immune cells showed constantly strong expression of Progranulin, only the number of these infiltrating cells was semiquantitatively assessed. Progranulin-immunoreactive immune cells were evaluated for their quantity in the lamina propria (1 = few, 2 = moderate, 3 = abundant). Therefore, the maximum score of immune cell-related expression of Progranulin was "3".

### Cell Culture and *in vitro *studies

AGS (CRL-1739) gastric cancer cells were purchased from American Type Culture Collection (ATCC). Cells were maintained in 25 cm^2 ^cell culture flasks (NUNC GmbH, Wiesbaden, Germany) in a cell incubator at 37°C and 5% CO_2 _using RPMI-1640 containing 10% FCS, 100 U/ml Penicillin, 100 μg/ml streptomycin and 100 μg/ml gentamycin (all reagents; PAA, Colbe, Germany).

Infection studies were performed using wildtype *H. pylori *strain purchased from ATCC (No. 43504). *H. pylori *was cultivated on selective agar plates (bioMerieux, Marcy I'Etoile, France) under microaerophilic conditions at 37°C for 2 days, and then resuspended in PBS (pH 7.4). Bacterial suspensions were adjusted based on optical density at 535 nm (OD = 1 corresponds to 1 × 10^9 ^bacteria). To ensure functional active bacteria, suspensions were microscopically inspected for shape and motility. After washing cells twice with medium without FCS and antibiotics, cells were infected with *H. pylori *at a "multiplicity of infection" of 50 in medium lacking antibiotics for 24 h.

For siRNA transfection, 4 × 10^5 ^cells were seeded in complete medium in 6-well plates and cultivated for 24 h. Cells were transfected with either SLPI-siRNA#1 (No: S100726383) or All-Stars™ negative siRNA control at a final concentration of 3 nM using HiPerfect™ transfect reagent as described by the manufacturer (all reagents, siRNA from Qiagen). Cells were cultivated in the presence of siRNA for another 48 hours at standard conditions, and then infected with *H. pylori *as described above.

After completing transfection and/or infection experiments, 0.8 ml of the cell culture medium was collected, centrifuged at 8.000 × g, and the supernatant stored in aliquots at -80°C for analysis. AGS cells were washed three-times with PBS (pH 7.4), and then harvested by PBS (pH 7.4) using a cell-scraper. Cells were washed once (8.000 × g, 4°C, 15 min) and resuspended in 1 ml PBS (pH 7.4). The sample was aliquoted (2 × 500 μl) into two Eppendorf tubes™ (Eppendorf AG, Hamburg, Germany), cells were obtained by centrifugation and the resulting pellets were stored at -80°C until analysis. Three individual experiments (each as duplicate) were performed for all experiments settings.

### Statistical Analysis

All data were entered into a database using the Microcal Origin™ 8.0G program package (Northhampton, MA, USA). Data are expressed as raw, median, mean ± standard deviations error (SD), or 95% CI (confidence intervals), if not stated otherwise. Non-parametric Kruskal-Wallis test and Mann-Whitney U test were applied for multiple and pairwise comparisons between groups, respectively. Immunohistochemical data were analyzed by One-way ANOVA (as global test for multiple testing) and LSD as post hoc analysis for pairwise comparisons if global test reached significant level. Correlation analysis was performed by Pearson test. All test were applied two-sided with a level of significance of P < 0.05.

## Results

### Expression of Progranulin in gastric mucosa in relation to *H. pylori *status and SLPI levels

**Figure 1:**
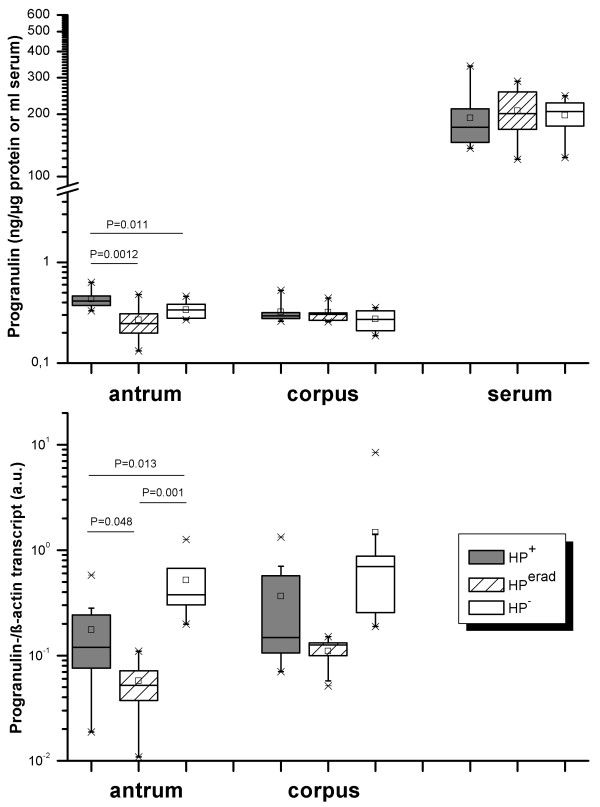
**Progranulin levels in gastric mucosa and serum in relation to *H. pylori-*status**. Data were stratified to location (antrum, corpus, serum) and *H. pylori *status as indicated by the legend and X-axes. Boxes represent the 25th, 50th and 75th percentile values (horizontal lines of the box) and means (squares). Significant differences were identified by Kruskal-Wallis (for multiple groups) and Mann-Whitney U test (pairwise comparisons) for antrum only; corresponding P-values are shown.

Progranulin gene expression and corresponding protein levels were identified in all mucosal samples from antrum and corpus as well as serum levels. As shown in figure 1, protein levels demonstrated normal distribution, while gene expression levels revealed skewed distribution. Therefore, we decided to apply nonparametric tests for both methodologies.

*H. pylori*-infected subjects had about 2-fold higher Progranulin protein levels (median: 0.43, range: 0.33-0.63 ng/μg protein) compared to levels after the successful eradication (median: 0.25, range: 0.25-0.46 ng/μg protein) or the unrelated *H. pylori-*negative group (median: 0.34, range: 0.27-0.46 ng/μg protein; p < 0.05) (Figure 1, upper panel). Progranulin protein levels in corpus mucosa (medians: 0.19 - 0.26 ng/μg) and serum samples (medians: 173 - 206 ng/ml) did not differ among the three groups (Figure 1, upper panel). Progranulin-mRNA amounts differed significantly in antrum among the three groups. As illustrated in figure 1 (lower panel), *H. pylori*-negative subjects revealed highest transcript amounts (median: 0.38, range: 0.2-1.3 a.u.), followed by the *H. pylori*-positive subjects (median: 0.17, range: 0.02-0.58 a.u.), and were lowest after eradication (median: 0.06, range: 0.03-0.07 a.u.). Similar results were obtained for corpus mucosa without reaching significance (Figure 1, lower panel).

**Figure 2:**
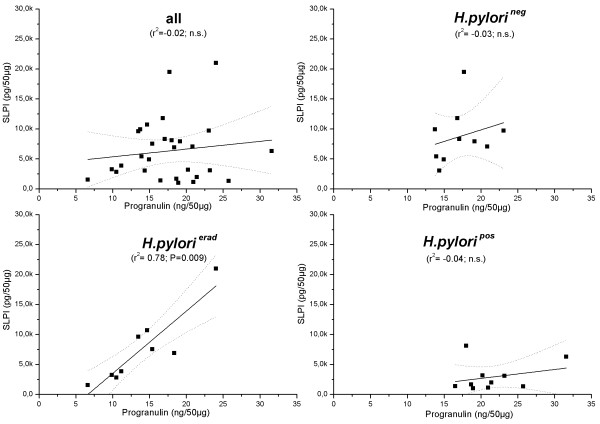
**Correlation analyses between antral levels of SLPI (Y-axes) and Progranulin (X-axes)**. Three groups and the combined analysis are indicated in the figure. Each dot represents one proband. Significant correlation and a non-significant trend (one outlier, marked with a cross) were observed for *H. pylori*-eradicated and - positive group.

To investigate a potential association between mucosal Progranulin and SLPI levels, correlation analysis was performed between both parameters. Please note that data concerning SLPI expression in these cohorts were published previously; therefore these data are not shown in detail in this study [20]. As illustrated in figure 2, a significant positive correlation was identified in eradicated subjects, whereas no correlation was seen in both other groups as well as in the combined data set. No correlations between Progranulin and SLPI were identified in corpus mucosa and serum of the three individual groups (data not shown).

### Immunohistochemical localization of Progranulin in the gastric mucosa

**Figure 3:**
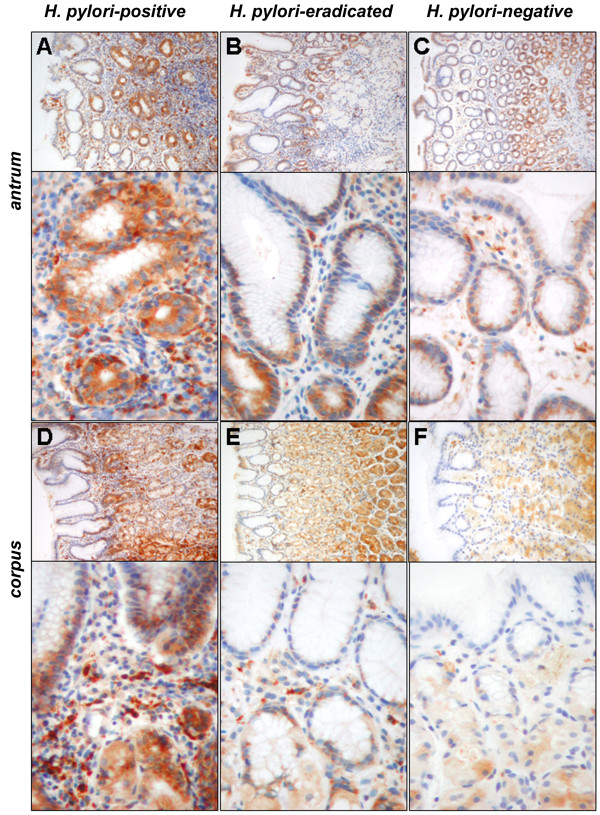
**Immunohistochemical detection of Progranulin in gastric antral mucosa**. Immunohistochemical stainings exemplarily illustrate Progranulin expression in biopsies from gastric mucosa of the antrum and corpus, respectively, of *H. pylori*-positive (A+D), -eradicated (B+E) and -negative (C+F) subjects as identified in figure. Expression was seen in a granular pattern evenly distributed to the epithelial cytoplasm of the glands and crypts and accentuated to the base of the surface epithelium. Enlargements of panel A+D demonstrate the Progranulin-expressing immune cells (mostly granulocytes) in the mucosa of an infected individual, whereas these cells are less abundant in corresponding samples of *H. pylori*-eradicated (panel B+E) and -negative individuals (panel C+F). Microscope: Zeiss Axioscope 50, camera: Nikon coolpix 990; enlargements: ×100, ×400.

**Figure 4:**
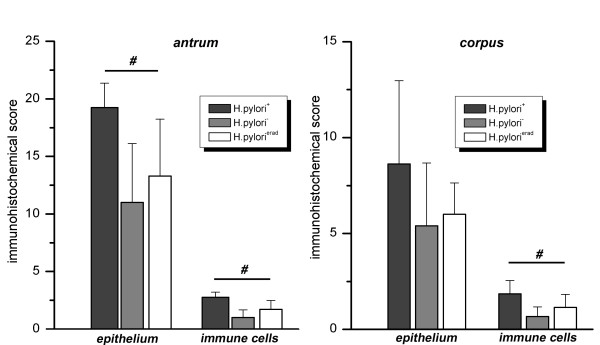
**Semiquantitative analysis of Progranulin expression in gastric mucosa**. Data illustrate semiquantitative immunoreactive scores (mean ± standard deviation) of Progranulin expression for gastric epithelium and immune cells (antrum: left panel; corpus right panel). Note that maximal scores are 24 and 3 for gastric epithelium and immune cells, respectively. The presence of significantly different scores among the three groups was performed by ANOVA test; significant differences are marked by "#".

As illustrated in figure 3, both epithelial and infiltrating immune cells contribute to the mucosal Progranulin expression. Immune cells (granulocytes, lymphocytes) showed constantly high expression of Progranulin except cells of lymphoid follicles. Higher numbers of Progranulin-expressing cells were associated with gastritis in *H. pylori*-infected subjects (Figure 3A+D). For the epithelium, strongest expression was observed in the gastric glands followed by the basis of the foveolae mainly in areas of dense inflammatory infiltrate. Surface epithelium between gastric pits showed weak or no expression of Progranulin. Semiquantitative scoring revealed significant higher expression scores of Progranulin for *H. pylori*-infected subjects compared to both other groups in antrum (Figure 4, left panel), whereas a tendency was observed for corpus (Figure 4, right panel). Furthermore, the number of infiltrating Progranulin-expressing immune cells was significantly higher in both antral and corpus mucosa of *H. pylori*-infected subjects (Figure 4).

### Expression of Progranulin and SLPI in epithelial AGS cells infected by *H. pylori*

**Figure 5:**
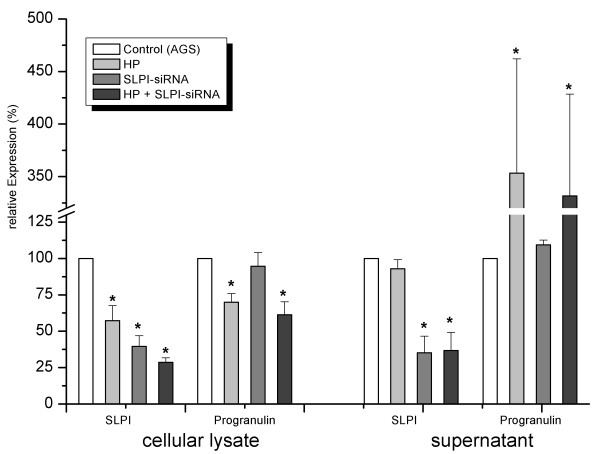
**Expression of Progranulin in relation to SLPI and *H. pylori *in AGS cells**. AGS cells were treated as indicated in the legend and explained in the section "Material and Methods". Data present relative values of 3 individual experiments (each with duplicates). SLPI and Progranulin levels were quantified by ELISA; control values (100%) were for SLPI: 64.3 ± 15.2 pg/50 μg and 452.4 ± 116.4 pg/ml and Progranulin 1.2 ± 0.09 ng/50 μg and 0.36 ± 0.1 ng/ml. Control transfection experiments using "all-star-negative"™ siRNA (Qiagen) as negative control showed a reduction of SLPI levels to 82.1 ± 6.3% and 82.3 ± 19.2% for lysate and supernatant, respectively (n = 3, each duplicates). Cells were transfected with siRNA, infected with *H. pylori *after 48 h and harvested after 72 h. Asteriks (*) illustrate significant changes in relation to corresponding control (two-sided paired T test, P < 0.05). Global test for multiple groups (ANOVA) was significant for all four groups (P < 0.05).

To investigate the regulatory link between SLPI and Progranulin, both molecules were investigated in relation to *H. pylori *infection and siRNA-mediated downregulation of SLPI expression in AGS cells. As demonstrated in figure 5, cellular SLPI levels were significantly reduced by 33%, 63%, and 81.3% by *H. pylori*, siRNA, and both factors, respectively. SLPI levels in the supernatant were strongly reduced (-65%) by siRNA, but not by *H. pylori *(Figure 5). The analysis of Progranulin levels in the identical samples, revealed no effect of SLPIsiRNA treatment. Both cellular (94.7 ± 9.4%) as well as secreted (109.4 ± 3.3%) Progranulin levels were similar to those of controls. *H. pylori*-infection was associated with elevated Progranulin level in supernatant (353 ± 109%), while cellular levels were found to be slightly reduced (70 ± 5.9%, P < 0.05). The combined effect of *H. pylori *and SLPI-siRNA approach resulted in similar changes (331 ± 97% and 61.3 ± 8.9% of Progranulin levels in supernatant and lysate, respectively (P < 0.05, Figure 5).

## Discussion

Here we demonstrate that (I) the *H. pylori *infection is associated with increased Progranulin levels in the antrum of infected subjects, and (II) that both epithelial and infiltrating immune cells contribute to this phenomenon. Furthermore, we provided evidence that (III) the upregulation of Progranulin seems to be independent of SLPI levels. Considering the central role of the elastase/SLPI equilibrium for the conversion of Progranulin to granulins [10] and the previously identified deregulation of elastase/SLPI expression in *H. pylori*-induced gastritis [21], we anticipated a negative correlation between SLPI and Progranulin for this disease. The *H. pylori*-induced reduction of mucosal SLPI levels resulted in higher elastase activities that were expected to degrade Progranulin leading subsequently to diminished mucosal Progranulin levels. In contrast to our working hypothesis (we expected a negative correlation between mucosal SLPI and Progranulin levels), we identified an increase of mucosal Progranulin levels in the antrum of *H. pylori-*infected subjects. Furthermore, correlation analyses revealed rather a trend or even a positive correlation between both proteins implying that the proposed regulatory link between SLPI and Progranulin is not present in this disease.

The fact that increased Progranulin levels were mostly restricted to antral mucosa (except immunohistochemical score of corpus glands) suggests an association of this upregulation with the degree of gastritis. As previously demonstrated, all probands presented antrum-predominant gastritis that was associated with moderate and severe activity scores reflecting the number of infiltrating granulocytes and lymphocytes [20]. As shown in immunohistochemical stainings of the study, immune cells were strongly positive for Progranulin and represent a major source of mucosal Progranulin levels in addition to gastric epithelial cells. Collectively, data of immunohistochemistry correspond to quantitative assessment of Progranulin by ELISA supporting the identified upregulation of Progranulin in *H. pylori*-infection.

Interestingly, *H. pylori*-negative subjects revealed significant higher *progranulin *transcript levels, which were associated with lower protein levels, compared to those of the *H. pylori*-positive and -eradicated group. The missing concordance between transcriptional and protein level is not easily explained and remains unclear. One potential explanation might be different regulatory mechanisms of Progranulin expression in gastric epithelial cells of *H. pylori*-negative subjects, who have been negative for the complete life compared to individuals after successful eradication therapy being without *H. pylori*-infection for several months only. As shown recently for mucosal infiltration and by the numbers of Progranulin-expressing immune cells in this study, samples from patients after eradication therapy contained still lymphocytes leading to slightly higher chronicity scores [20] or slightly increased Progranulin scores compared to *H. pylori*-negative subjects. Since in *H. pylori*-positive subjects, two major Progranulin-expressing cell types (epithelial and immune cells) are simultaneously present, *Progranulin *transcript levels can not be assessed individually for each cell type. Despite the missing concordance between protein and transcript levels, it should be emphasized that the mucosal levels of Progranulin were found to be significantly upregulated in *H. pylori*-infected subjects.

The results obtained in the AGS cell model do partially not correspond to the *ex vivo *findings. While *ex vivo *data demonstrated an upregulation of Progranulin by *H. pylori*, in the AGS cell model, only the concentration of Progranulin in the supernatant was strongly induced, whereas the cellular expression, analyzed in the lysate, was decreased. There are several aspects that might explain these disconcordant results. In AGS cells, both the intracellular and secreted proportion of Progranulin was separately analyzed. Since in *ex vivo *analysis, both compartments can not be differentiated, the increased Progranulin levels in antral mucosa might reflect both increased secretion and changes in epithelial Progranulin expression. Second, *ex vivo *analysis is performed on complex samples including epithelial and immune cells, whereas the *in vitro *model only mirrors the direct interaction of *H. pylori *to epithelial-derived AGS cells. Third, analyzing the Progranulin expression after 24 hours represents the effects of an acute infection, whereas changes in mucosal biopsies can be considered as long-term effects of an chronic infection that are in a "steady-state". Despite these limitations, data from the *in vitro *model allow the conclusion that a down-regulation of epithelial SLPI expression (either by *H. pylori *or siRNA) does not affect the expression of Progranulin in AGS cells. Owing to the low molecular weight of granulins, no method is currently suitable to analyze quantitatively the levels of the Progranulin-derived degradation products. Therefore, no statement can be made concerning the equilibrium between Progranulin and granulins in gastric mucosa that might hypothetically be shifted towards granulins even the Progranulin levels are upregulated. Furthermore, it is of note that SLPI is not the only serine protease inhibitor expressed in the gastric mucosa. Recently, we identified elevated alpha-1 protease inhibitor (A1-PI) levels in the mucosa of *H. pylori*-infected individuals [30]. Since A1-PI can inhibit elastase to a similar extent as SLPI [7], a compensatory mechanism is another potential explanation, while Progranulin is elevated, although SLPI levels are strongly diminished in relation to *H. pylori *infection.

The observed association of induced Progranulin levels in context to *H. pylori *infection and its associated gastritis does not allow functional conclusions whether the upregulation has an active regulatory role for the inflammatory process, or it merely reflects the inflammatory conditions of the underlying gastritis. Keeping in mind that Progranulin acts as epithelial growth factor in other diseases [28,29], it is tempting to speculate that the upregulation of Progranulin in *H. pylori-*associated gastritis might be involved in mucosal healing of gastric erosions/ulcers induced by this infection. But at this moment, this remains purely speculative since no functional data are available.

## Conclusions

Taken together data from *in vitro *and *ex vivo *analysis, we can conclude that the proposed regulatory link between SLPI and Progranulin expression seems to be of no or low relevance in context to the *H. pylori *infection. Furthermore, we provide evidence that Progranulin is another molecule the expression of which is upregulated in relation to this infection.

## Competing interests

The authors declare that none of them has financial interests in context to this study. This work was supported by the Deutsche Forschungsgemeinschaft (WE-2170/8-1).

## Authors' contributions

TW was involved in the conception and design of the study, analyzing and interpreting data, writing the manuscript and revision of the final version. DK performed immunohistochemical stainings, corresponding semiquantitative analysis and participated in writing the draft. CS and DS performed *in vitro *studies on AGS cells and the corresponding assessment and analysis of Progranulin expression in these samples. GT enrolled the patients groups, performed endoscopic evaluation including sampling biopsies, and contributed in writing the draft. PM was involved in the conception and design of the study, and revised the manuscript for important intellectual content. All authors read and approved the final manuscript.

## Pre-publication history

The pre-publication history for this paper can be accessed here:

http://www.biomedcentral.com/1471-230X/11/63/prepub
